# Climate and habitat type interact to influence contemporary dispersal potential in Prairie Smoke (*Geum triflorum*)

**DOI:** 10.1002/ece3.11231

**Published:** 2024-04-15

**Authors:** Lauren L. Sullivan, Zoe M. Portlas, Kelsey M. Jaeger, Mercedes Hoffner, Jill A. Hamilton

**Affiliations:** ^1^ Department of Plant Biology Michigan State University East Lansing Michigan USA; ^2^ Kellogg Biological Station Michigan State University Hickory Corners Michigan USA; ^3^ Ecology, Evolution and Behavior Program Michigan State University East Lansing Michigan USA; ^4^ Division of Biological Science University of Missouri Columbia Missouri USA; ^5^ Department of Ecology, Evolution and Behavior University of Minnesota Saint Paul Minnesota USA; ^6^ Department of Biological Sciences North Dakota State University Fargo North Dakota USA; ^7^ Department of Plant Biology University of Vermont Burlington Vermont USA; ^8^ Department of Ecosystem Science and Management Pennsylvania State University University Park Pennsylvania USA

**Keywords:** common garden, fragmentation, gene flow, *Geum triflorum*, isolation, local adaptation, natural populations, WALD model

## Abstract

Understanding dispersal potential, or the probability a species will move a given distance, under different environmental conditions is essential to predicting species' ability to move across the landscape and track shifting ecological niches. Two important drivers of dispersal ability are climatic differences and variations in local habitat type. Despite the likelihood these global drivers act simultaneously on plant populations, and thus dispersal potential is likely to change as a result, their combined effects on dispersal are rarely examined. To understand the effect of climate and varying habitat types on dispersal potential, we studied *Geum triflorum*—a perennial grassland species that spans a wide range of environments, including both prairie and alvar habitats. We explored how the climate of the growing season and habitat type (prairie vs. alvar) interact to alter dispersal potential. We found a consistent interactive effect of climate and habitat type on dispersal potential. Across prairie populations, an increased number of growing degree days favored traits that increase dispersal potential or the probability of dispersing farther distances. However, for alvar populations, dispersal potential tended to decrease as the number of growing degree days increased. Our findings suggest that under continued warming, populations in prairie habitats will benefit from increased gene flow, while alvar populations will become increasingly segregated, with reduced potential to track shifting fitness optima.

## Graphical abstract

We explored how the dispersal traits of *Geum triflourm* (Prairie Smoke), a perennial herb, changed with habitat type and climate. In isolated alvar habitats, *G. triflorum* seeds tend to disperse longer distances under shorter growing seasons and shorter distances under longer growing seasons. We found the reverse was true when *G. triflorum* was growing in continuous prairie populations. This was true for both (a) equal wind conditions and (b) when wind in prairies was increased relative to alvars.

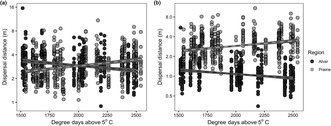

## 1 INTRODUCTION

Seed dispersal, or the movement of offspring away from the source parent plant, provides the opportunity for migration (Zobel et al., 2010), gene flow (Sexton et al., 2009), range shifts (Davis & Shaw, 2001; Hargreaves et al., 2015), and spatial tracking of favorable environmental conditions (Brehm et al., 2019; Edelaar & Bolnick, 2012)—all of which allow for species persistence despite local extirpation (Tilman, 1994). Indeed, dispersal evolves even in the simplest systems with homogenous environments (Hamilton & May, 1977) and is especially important for sessile organisms, such as plants, that have limited opportunities during their life cycle for movement (Beckman & Sullivan, 2023). In heterogeneous habitats, environmental conditions are an important driver of plant dispersal ability, which can manifest locally through differences in physical properties of the environment (e.g., soil depth, habitat structure, fragmentation) as well as in a more broad‐scale way through the climate in the year of reproduction (Hernandez et al., 2023). When the impact of multiple environmental factors are considered synergistically on dispersal in theoretical studies, strong effects on plant movement are often found, with factors like fragmentation and climate change both slowing dispersal (Renton et al., 2012, 2013). However, there is a dearth of empirical studies that directly examine how local environmental differences and broad‐scale climate concomitantly alter plant dispersal. In an era of multiple anthropogenic global changes that simultaneously act on plant populations, it is imperative to determine how the local environment and climate singly and jointly influence plants' dispersal ability. Here, we focus on species that disperse their seeds by wind (anemochory), as those species are likely to be sensitive to environmental change (Beckman & Sullivan, 2023).

Climate is a key factors that influence seed dispersal (Hernandez et al., 2023), and an increasing thermal environment can have both positive and negative effects on dispersal capacity (Corlett & Westcott, 2013), with some plants actively controlling dispersal distance based on temperature (Seale & Nakayama, 2020). In some cases, warming temperatures and associated aridity can increase seed release height (Bjorkman et al., 2018; Teller et al., 2014), which has direct impacts on increasing potential dispersal distance (Thomson et al., 2011). This increase in dispersal can be exacerbated when these warmer temperatures change the speed, direction, and turbulence of wind conditions that further promote long‐distance dispersal (Kling & Ackerly, 2020; Kuparinen et al., 2009). However, for some species, increasing temperature can have the opposite effect on dispersal. There is evidence that species that produce heteromorphic seeds will disproportionately produce seeds with reduced dispersal potential under warmer environments to persist if changed local environments are suitable, thus producing a larger proportion of seeds that travel shorter distances (Arshad et al., 2019; Lenser et al., 2016). To predict how dispersal might change as climate continues to warm there is a need to understand the degree to which species‐specific dispersal potential is sensitive to climatic variation.

The local environment a plant grows in can also affect dispersal potential (Cheptou et al., 2008; Damschen et al., 2014; Hernandez et al., 2023). Deeper soils with more available nutrients can allow plants to grow taller (Bernard‐Verdier et al., 2012; Dickson et al., 2014), which can increase dispersal ability (Thomson et al., 2011). When similar plant species exist in different habitat types, that vary in soil depth, as well as nutrient and water availability, plants on shallower soils tend to decrease in height relative to those on deeper soil (Harikrishna et al., 2005; Tardella et al., 2017). One way to study the effects of the local environment on dispersal traits is to select environments that exist in extremes. For example, grassland habitats tend to have relatively deep soil, but a subset of herbaceous species that live in grasslands can also live in similar habitats with much shallower soils (e.g., alvars, glades, serpentine, rocky outcrops) (Hamilton & Eckert, 2007; Nelson, 1987; Visioli et al., 2019). By comparing dispersal potential of species that can live in both habitat types, we can determine how the suite of local environmental conditions that drive these habitat differences alter plant movement.

The simultaneous impacts of both climate and local environmental differences driven by habitat type on dispersal could result in either concomitant or interactive outcomes. When developing predictions for these interactive effects, results are likely to be highly dependent on the species in question, its evolutionary history, and its current environmental conditions (Beckman et al., 2020). If a species' dispersal ability responds in concert with respect to both habitat type and increasing temperatures, then you would expect the species to either increase or decrease its dispersal ability as these factors change. However, more complicated outcomes can arise when interactive effects occur, as Delattre et al. (2013) observed in a species of butterfly. When a species' dispersal ability responds differently to climatic factors in different types of environments, the resulting interactive effects can lead to more nuanced environment‐specific responses. Interactive effects are nearly impossible to tease apart without considering both factors directly and simultaneously, especially when interactions are nonadditive (Galic et al., 2018; Harpole et al., 2011). Thus, it is imperative to quantify the relationship between climate and habitat type simultaneously on dispersal ability in order to predict changes to these selective pressures (Fahrig, 2017; Parmesan & Yohe, 2003).

Here, we examine how climate and habitat type interact to impact dispersal ability using a species that spans a broad climatic gradient across prairie and alvar habitat types, *Geum triflorum*, Prairie Smoke (Rohrer, 1993). We examine contemporary differences in population‐level dispersal trait values and use these to estimate changes in dispersal potential. Wind‐dispersed plant species, like *G. triflorum*, have evolved multiple mechanisms that promote movement by wind, including structures such as plumes, wings, or samaras that increase time aloft and in turn increase dispersal distances (Greene & Johnson, 1990; Lentink et al., 2009; Varshney et al., 2012). Specifically, we aim to determine if climate (specifically, the number of growing degree days above 5°C) and habitat type (either isolated alvars with shallow soils or more continuous prairie habitats with deeper soils) have positive, negative, or interactive effects on contemporary dispersal trait variation including shape, mass, and falling speed that collectively influence dispersal potential. We measured dispersal traits from 100 *G. triflorum* populations across its range, including populations from both alvar and prairie habitats. Using these traits, we parameterized dispersal models to determine dispersal potential of individuals within each population. We found that climate and habitat type additively interact to influence dispersal trait variation, and in turn dispersal potential of *G. triflorum*. In prairie habitats, increasing the number of growing degree days increased dispersal ability, whereas in alvar habitats, increasing the number of growing degree days reduced dispersal ability. These results indicate the importance of considering the interaction between climate and the habitat type on dispersal potential and provide important hypotheses for future experiments to test the causal factors that drive a species' ability to spatially track shifting fitness optima under global change.

## 2 MATERIALS AND METHODS

### 2.1 Study species

**FIGURE 1:**
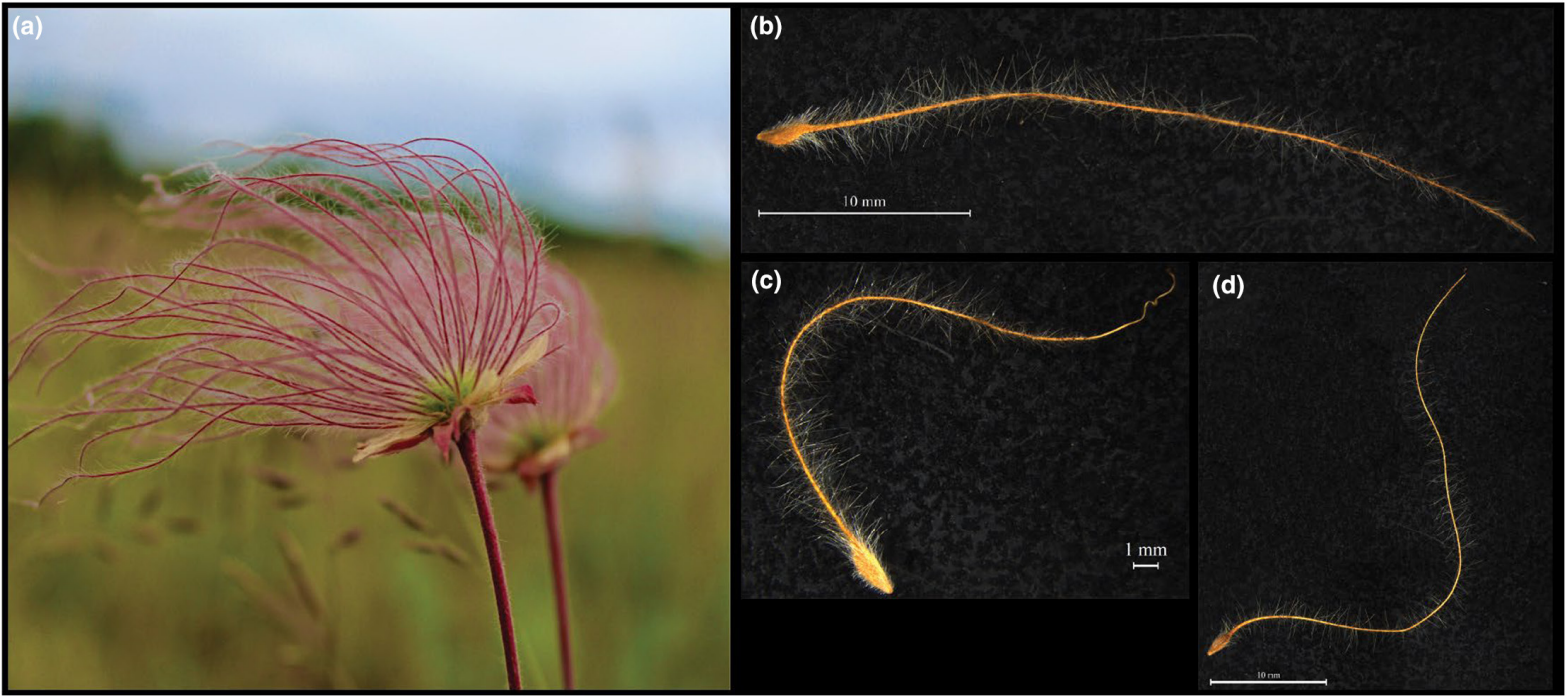
*Geum triflorum* (a) fruiting head and (b–d) diaspores. Each diaspore contains an achene that is connected to a densely wooly style that remains intact at the time of dispersal. These hairs help slow the rate of falling and thus help increase the dispersal ability of these diaspores. Panels b–d represent the range of diaspore shapes and sizes.

*Geum triflorum* (Pursh), or Prairie Smoke (Rosaceae), is an herbaceous perennial distributed across much of northern North America (US and Canada) and in the southwestern United States and California (Gajewski, 1958; Rohrer, 1993). Inflorescences typically have three nodding flowers that become erect at seed set. The sessile infructescence contains multiple achenes, each connected to a densely wooly style that remains intact during dispersal (Figure 1a). This structure, hereafter termed “diaspore,” includes both the achenes and style (Figure 1b–d). These styles likely promote wind dispersal by increasing the time‐aloft for diaspores following release from the maternal plant (Greene & Johnson, 1989, 1990).

### 2.2 Sampling

**FIGURE 2:**
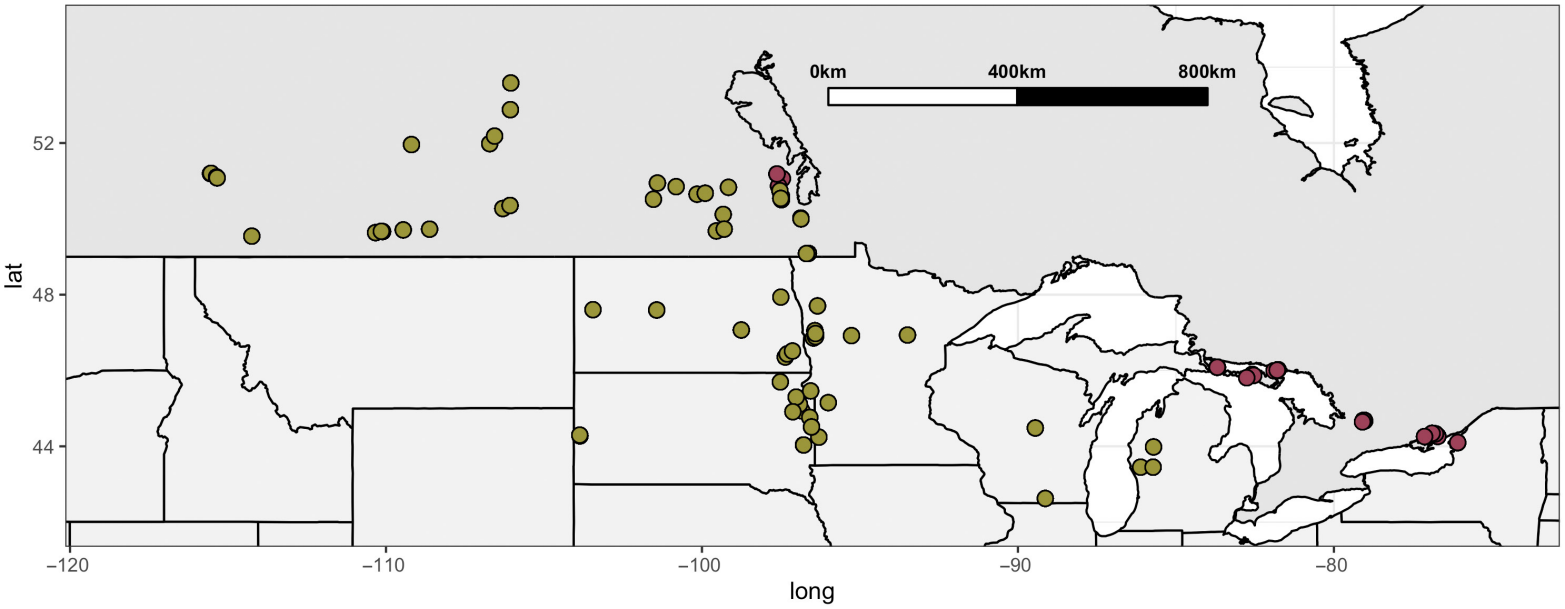
*Geum triflorum* populations sampled for the study across the United States and Canada. Yellow dots indicate prairie populations, and red dots indicate alvar populations. All populations are within a similar range of DD5 (number of growing degree days over 5°C).

We collected individual seed heads from populations of *G. triflorum* in 2002, 2003, 2015, and 2016. These populations were defined as distinct groups of plants separated from other populations by at least 1 km. Individual seed heads were collected from between 40 and 150 plants along a 100 m transect in 2002, 2003, and 2015 and along a 1000 m transect in 2016. See Hamilton and Eckert (2007) for detailed sampling methods. In total, we sampled 60 continuous prairie populations and 40 isolated alvar populations (ecoregional types described in Section 2.3 below, Figure 2).

### 2.3 Habitat type

To determine how differences in habitat type alter dispersal traits, we sampled *G. triflorum* populations across its range throughout the midwestern prairies of North America, and across disjunct populations geographically isolated on limestone barrens known as alvar habitats, which are distributed throughout the Great Lakes region and into Manitoba, Canada. The species occurs in four of seven alvar regions across the Great Lakes (Catling & Brownell, 1995; Reschke et al., 1999); the Napanee Plain, the Carden Plain, Western New York, and Manitoulin Island; as well as alvar habitats on Drummond Island, Michigan, and northern Manitoba. *G. triflorum's* prairie range extends from west of the Great Lakes across the Rocky Mountains, covering much of the Great Plains of Canada and the United States.

In general, prairies and alvars differ in both abiotic and biotic conditions. Their edaphic environments differ with alvars having thin layers of soil over limestone and exposed areas of bare rock (Belcher et al., 1992; Catling & Brownell, 1995; Hamilton & Eckert, 2007), contrasting with prairies which tend to have deep soils which allow for deeper rooting systems (Anderson et al., 2006; Volk et al., 2022). The plant communities on alvars and prairies are also different. Alvars host a large number of species that are disjunct from their core distributions (northern, southern, western disjuncts), in addition to some endemics and persist as islands of open habitat within a broader matrix of conifer‐dominated boreal forest (Brownell & Riley, 2000; Catling & Brownell, 1995). In contrast, prairies are largely open, and comprised of mixed, short, or tallgrass systems that are subject to climatic extremes (Samson & Knopf, 1994). In general, local densities of *G. triflorum* tend to be slightly higher in alvars than prairies.

Historic connectivity also varies between prairie and alvar populations (Hamilton & Eckert, 2007), which has implications for the evolution of dispersal traits. Prairie populations have historically experienced higher levels of genetic connectivity (Hamilton & Eckert, 2007), but extreme anthropogenic fragmentation across its range over the last century (Lark et al., 2018; Samson & Knopf, 1994; Wright & Wimberly, 2013) has led to increased isolation (Sullivan et al., 2021; Wimberly et al., 2018). In contrast, historically isolated alvar populations are not only disjunct from the main contiguous range across the midwestern prairies but also are isolated from each other. Grassland species likely colonized alvar habitats (Hamilton & Eckert, 2007), however, it is likely that subsequent range contractions isolated grassland species, including *G. triflorum*, on alvar habitats (Catling & Brownell, 1995; Hamilton & Eckert, 2007). Genetic data indicate that alvar populations have a subset of the genetic variation found in prairie populations, and within the same geographic distance, alvar population pairs are significantly more genetically differentiated from each other than prairie population pairs (Hamilton & Eckert, 2007). Thus, contemporary differentiation between prairie and alvar populations likely reflects a combination of colonization history and contemporary gene flow, stochastic processes associated with shifts in population demography, and changing selective processes leading to adaptation across regional environments.

### 2.4 Climate

To determine how climate influences dispersal traits of *G. triflorum*, we used year of collection, latitude, longitude, and elevation from sampling locations as inputs into ClimateNA (Wang et al., 2016) to estimate climate variables associated with geographic provenance. We estimated climatic variables associated with the year of seed collection to control for temporal variation in the maternal environment that may influence development across our multiple years of sampling, and because the climate associated with the year of collection represents the environmental conditions that corresponds with diaspore development. For each population, we were able to estimate annual climate variables including many related to precipitation and temperature. In order to reduce redundancy and account for correlation across climate variables, we performed a principal component analysis (PCA). We found temperature variables (number of growing degree days above 5°C, mean annual temperature, frost‐free period, etc.) largely loaded on the PC1 axis and explained 51% of the variation, while variables associated with water availability (mean annual precipitation, climatic moisture deficit, annual heat moisture index, etc.) largely loaded on PC2 axis and explained 23.6% of the variation. To simplify our analyses, we described climate using the “number of growing degree days above 5°C” variable (hereafter referred to as DD5) as it loads highly on PC1 representing much of the temperature variability spanning our populations. Specifically, DD5 is defined as the sum of days when the mean temperature was above 5°C and reflects the time of “active growth” for Prairie Smoke populations (Thibault et al., 2020). Furthermore, it is a biologically meaningful climate variable as it reflects the required heat sum associated with the onset of growth (Beaubien & Hamann, 2011). Development is predicted to be particularly sensitive to climate change (McGinn & Shepherd, 2003) as shifts in DD5 for spring perennials, such as *G. triflorum*, have substantial influence on the timing and duration of the growing season (Beaubien & Hamann, 2011; Kulbaba et al., 2023; Volk et al., 2022; Whittet et al., 2017). We note here that both prairie and alvar populations fell within a similar latitudinal range, therefore values of DD5 are largely independent of photoperiod (e.g., each habitat type on the whole had overlapping distributions of DD5).

### 2.5 Dispersal trait measurements

To measure dispersal traits, we randomly selected three to five individual seed heads per population, each seed head representing one maternal family. We then measured three types of dispersal traits on all diaspores (Figure 1), including mass, morphology, and terminal velocity (or falling speed). Pooling five individual diaspore measures per maternal family, we first estimated total diaspore mass. We weighed both the achene and style separately to the nearest 0.0001 g. Following this, we examined morphological variation for each of five individual diaspores per maternal family. We photographed diaspores on a 5 mm^2^ grid using a Leica DM2500 dissecting microscope for all morphological measurements. We placed a glass sheet atop each diaspore to uniformly flatten the dispersal structures. All measurements were made using ImageJ (Schneider et al., 2012). We measured the total length of the style and achene to the nearest 0.001 mm, and the achene area to the nearest 0.001 mm^2^. We measured the total length of the style and achene as the entire path length. We calculated achene area using the length and width measured at the longest and widest points, respectively. In addition, because diaspore length represented the path length of each seed, but sometimes seeds were folded, and not straight (Figure 1c vs. 1b), we also calculated diaspore shape index, which is a measure of 2D shape of each diaspore. This was calculated as an area, using the length of the longest dimension, and the perpendicular longest length. Finally, to measure terminal velocity, we dropped up to five individual intact diaspores per maternal family through a modified 10 cm wide PVC tube with two sets of light sensor arrays spaced 57 cm apart that record when the diaspores passed through both sets of arrays. We recorded the time it took the diaspores to cross this distance as the terminal velocity. For a full description of the terminal velocity measurement device and setup, see Sullivan et al. (2018). Diaspores were dropped from a height of 1 m to ensure they reached terminal velocity. For terminal velocity measurements we used data from 26 prairie populations and 17 alvar populations reflecting contemporary collections from 2015 to 2016.

In order to translate terminal velocity measurements into dispersal ability, we used the WALD model (Katul et al., 2005), which has been empirically validated for grassland species (Soons et al., 2004; Sullivan et al., 2018), to calculate the dispersal distances traveled by the farthest 1% of dispersers. To do this, we followed Sullivan et al. (2018) and used plant traits including height at diaspore release and terminal velocity to parameterize the estimated dispersal kernel for each diaspore. We estimated pooled height at diaspore release for each habitat type as the average height from 53 prairie and 18 alvar flowering stems of *G. triflorum* (measured from rosette to base of sepals) from herbaria specimens provided by the University of Minnesota (MIN), University of Manitoba (WIN), the Canadian Museum of Nature (CAN), and the Agriculture and Agri‐Food Canada National Collection of Vascular Plants (DAO). Analysis of variance indicated no difference in height between plants in the alvars and prairies (F_1_ = 0.461, *p* = .499). We estimated the canopy height to be 0.2 m for both regions at the time of *G. triflorum* seeding, as this is early in the season and many plants are still short. Finally, we parameterized wind values from weather station readings near Moorhead, MN. We used a grand average of 7‐day wind averages collected approximately every 4 days during the month of June in 2017 and 2018 as this is when the majority of *G. triflorum* diaspore dispersal occurs. We used these data to estimate wind parameters in prairie systems, and assumed the wind to be either equivalent between the two habitat types, or 50% of this average value for alvars, as these isolated habitats are surrounded by forest trees, which can substantially reduce wind effects (Damschen et al., 2014). Dispersal kernels were modeled for each individual diaspore within each habitat type, following which we extracted the predicted distance traveled if the diaspore achieved its long‐distance dispersal potential (Higgins et al., 2008).

### 2.6 Statistical analysis

To determine how climate and habitat type (alvar vs. prairie) interact to influence dispersal traits of *G. triflorum*, we explored our response variables with mixed effect models. We ran three separate models, one for each of the three categories of dispersal traits, including mass, morphology, and terminal velocity. For these dispersal trait models, all trait variables (response variables) were transformed using a square root transformation to meet the assumption of normality. For these models, the fixed effects included the interaction between habitat type (prairie vs. alvar) and the log DD5 estimated from the growing season in which diaspores were collected. Our random intercepts included family nested within population (except for the mass model that only had one value per family, thus we simply included a random effect for population), and an additional random intercept term for year of data collection. We note that year effects explain about 20% of the variation for these models, but opt to leave this in the random effects because it is not the main focus of our study. This appears to correlate strongly with precipitation patterns.

Additionally, to determine the effect of climate and habitat continuity on dispersal potential, we analyzed the log of the distance traveled by the farthest 1% of dispersing individuals using another mixed effects model. Here, our fixed effects were the interaction between habitat type and log DD5. Our random effects included an intercept of family nested within population, and a year intercept.

We used R v4.2.0 (R Core Team, 2022) to conduct mixed effect models with the lmer() function in the lme4 package (Bates et al., 2015), and used the lmerTest package (Kuznetsova et al., 2014) to extract *p*‐values. We also used the MuMIn package (Barton, 2018) to determine the contributions of the fixed and random effects using *r* ^2^ values. Data and code for analyses and figure creation can be found at Dryad: https://doi.org/10.5061/dryad.3bk3j9kt0.

## 3 RESULTS

### 3.1 Mass measurements

**FIGURE 3:**
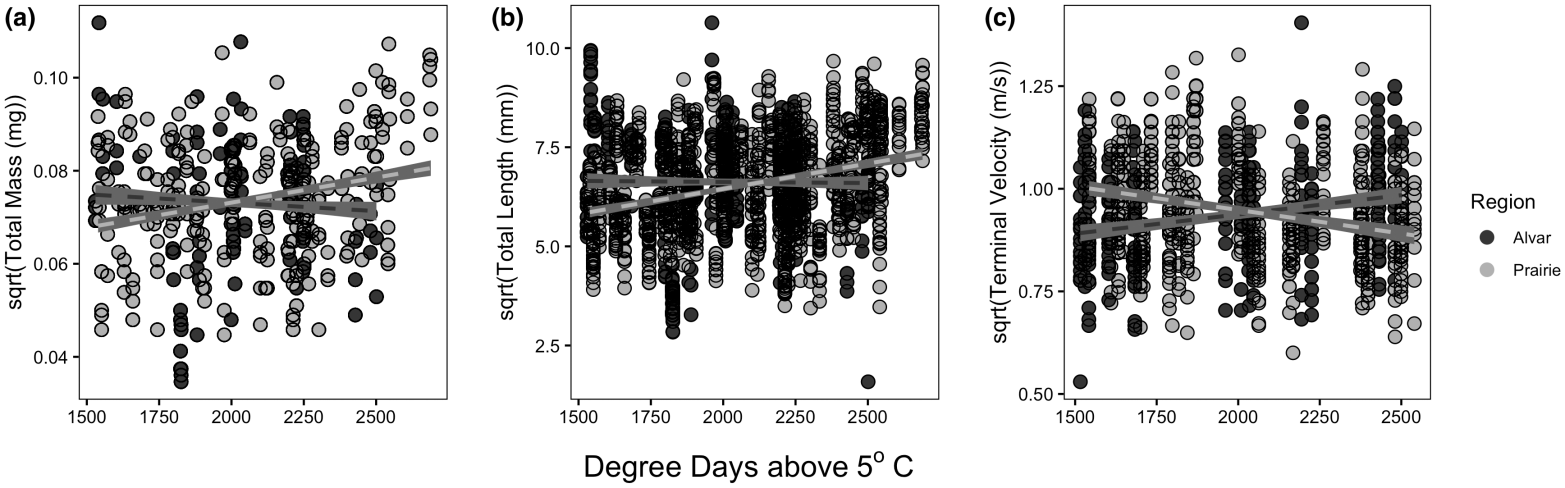
Relationship between diaspore traits and degree days above 5°C (DD5) in both prairie (light gray) and alvar (dark gray) regions. Total diaspore mass (a) showed a significant interaction between habitat type (prairie vs. alvar) and climate—as the growing season gets longer (increased DD5), diaspore mass increased in prairies, while alvar diaspore mass decreased slightly. Diaspore length (b) shows a significant interaction between habitat continuity and climate. As the season gets longer (increased DD5), in prairies diaspores get longer, and in alvars, there is little change. Terminal velocity (c) showed a strong interaction between habitat continuity and climate. As the length of the growing season increased (increased DD5), in alvars the terminal velocity of a diaspore increased, in prairies, the terminal velocity decreased. This indicates that diaspores have the potential to travel further distances in prairies than in alvars, in longer seasons, but travel further in alvars when the season is shorter.

We found a significant interaction between habitat type (alvar vs. prairie) and climate for total diaspore mass (Table 1A, *p =* .021), where fixed effects explained 4.6% of the variance, and fixed and random effects together explained 65.6% of the variance. As the length of the growing season increases, prairie diaspores become heavier, while alvar diaspores become lighter (Figure 3a).

**TABLE 1:** Mixed effects model results for (A) diaspore mass, (B) total diaspore length, (C) terminal velocity, and (D) long‐distance dispersal potential.

	Estimate	Std. error	*df*	*t*‐Value	*p*‐Value
(A) Diaspore mass					
Intercept	0.160	0.086	103.42	1.861	.066
Habitat type (Prairie)	−0.231	0.100	103.15	−2.331	.022
DD5	−0.011	0.011	103.35	−1.015	.313
Habitat type (Prairie):DD5	0.031	0.013	103.34	2.347	**.021**
(B) Total Diaspore length					
Intercept	13.583	7.505	100.75	1.810	.073
Habitat type (Prairie)	−26.853	8.674	100.16	−3.096	.003
DD5	−0.927	0.990	100.75	−0.938	.351
Habitat type (Prairie):DD5	3.543	1.000	100.36	3.093	**.003**
(C) Terminal velocity					
Intercept	0.065	0.757	42.95	0.086	.932
Habitat type (Prairie)	2.918	0.983	41.15	2.968	.005
DD5	0.113	0.100	42.97	1.132	.264
Habitat type (Prairie):DD5	−0.380	0.130	41.22	−2.929	**.006**
(D) Dispersal potential—equal wind					
Intercept	13.953	6.656	43.34	2.096	**.042**
Habitat type (Prairie)	−29.847	8.657	41.43	−3.448	**.001**
DD5	−1.411	0.879	43.37	−1.605	.116
Habitat type (Prairie):DD5	3.910	1.142	41.51	3.424	**.001**
(E) Dispersal potential—50% wind difference					
Intercept	3.373	6.079	43.30	0.555	.582
Habitat type (Prairie)	−18.940	7.917	41.26	−2.392	.021
DD5	−0.279	0.802	43.33	−0.348	.730
Habitat type (Prairie):DD5	2.736	1.044	41.35	2.620	**.012**

Bold values indicate p < 0.05 considered as statistically significant.

### 3.2 Morphology measurements

We found a significant interaction between habitat type (alvar vs. prairie) and climate on total diaspore length (seed plus style length) (Table 1B, *p* = .003), with fixed effects explaining 7.74% of the variation, and fixed and random effects together explaining 80.8% of the variation. This interaction suggests as growing season increases, diaspore length increases in prairies, but changes little in alvars (Figure 3b). We did not find a significant effect of habitat type or climate on diaspore area or diaspore shape index.

### 3.3 Terminal velocity measurements

We also observed a significant interaction between habitat type (alvar vs. prairie) and climate for terminal velocity of the diaspores (Table 1C, *p =* .006), with fixed effects explaining 8.9% of the variance, and fixed and random effects together explaining 55.4% of the variance. Longer growing seasons predict reduced terminal velocities in the prairies (i.e., diaspores fall slower), and greater terminal velocities in the alvars (i.e., diaspores fall faster) (Figure 3c).

We note for these three traits, the fixed effects explain a relatively small amount of variance, with much left to be explained. This is due in part to large sample size, and much of the variance being explained by the maternal effects and site and year differences. We encourage interpretation of our fixed effects with appropriate caution.

### 3.4 Estimated dispersal distances

**FIGURE 4:**
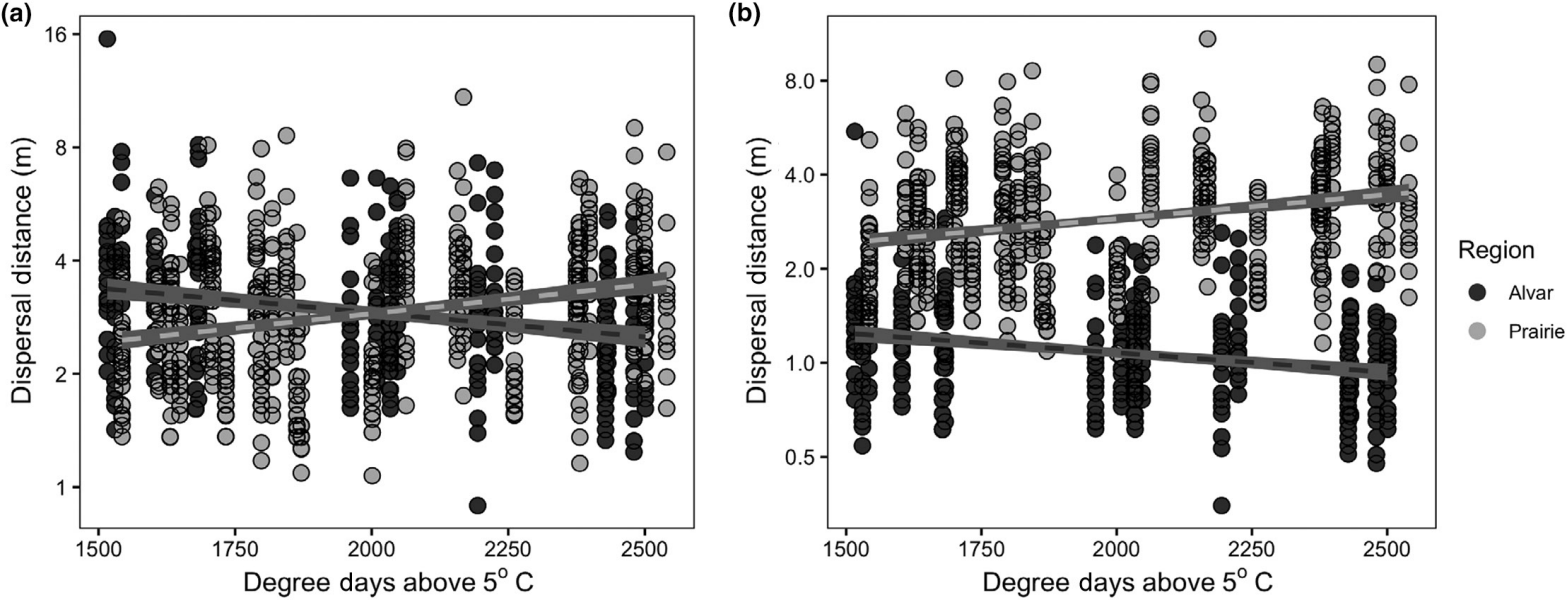
When using the WALD model to estimate dispersal distances for *Geum triflorum* individuals, we found a significant interaction between habitat type (prairie vs. alvar) and climate. As the length of the growing season increased (increased degree days above 5°C), in alvars (dark gray) the potential long‐distance dispersal ability (the distance traveled by the farthest 1%) of a diaspore increased, in prairies (light gray) the long‐distance dispersal ability increased. We ran these models under (a) equal wind conditions between prairies and alvars, and (b) a 50% reduction in wind speeds in alvar habitats compared with prairies.

We found that the interaction between habitat type (alvar vs. prairie) and climate significantly altered potential long‐distance dispersal of *G. triflorum* (Table 1D,E). When wind conditions were equal between prairie and alvar habitats, we found a significant interaction between climate and habitat type (*t* = 3.91, *p =* .001) with fixed effects explaining 6.7% of the variance, and fixed and random effects together explaining 39.6% of the variance. Longer growing seasons predicts increased long‐distance dispersal potential in the prairies, and reduced long‐distance dispersal potential in the alvars (Figure 4a). When wind conditions were 50% slower in alvar habitats, which is a reasonable assumption given alvars are predicted to have lower average wind speeds relative to prairie environments (Damschen et al., 2014; Schaefer & Larson, 1997), we found a similar significant interaction between climate and habitat type (*t* = 2.74, *p* = .012), with fixed effects explaining 43.2% of the variance, and fixed and random effects together explaining 66.0% of the variance. While the results are qualitatively similar, the differences in dispersal potential are much larger under the 50% wind reduction scenario (Figure 4b).

## 4 DISCUSSION

Here we demonstrate that climatic conditions and habitat type can interact to influence dispersal trait variation and potential dispersal ability. We found that with the accumulation of growing degree days, *Geum triflorum* exhibited increased dispersal potential in prairie habitats. In contrast, isolated alvar populations exhibited reduced dispersal potential as the number of growing degree days increased. Similar to the results of Delattre et al. (2013), these observational data suggest the relationship between movement, habitat type, and climate can be complex and interdependent. These results have the potential to predict the capacity by which *G. triflorum* will be able to track shifting fitness optima in rapidly changing environments via dispersal across varying environmental conditions (Davis & Shaw, 2001; Kokko & López‐Sepulcre, 2006). We encourage experimentally‐based examination of the interplay between climate and habitat type and its impact on dispersal potential across species, especially those of conservation concern, in order to predict shifts in gene flow and genetic connectivity across dynamic landscapes.

Our results suggest variance in dispersal potential is at least partly driven by the environment. For populations on alvars, which experience predictable seasonal variation from flooding to desiccation, in a longer growing season (e.g., more growing degree days above 5°C), limited access to water as the season progresses may impact development (Hamilton & Eckert, 2007; Volk et al., 2022). We find alvar populations of *G. triflorum* use shorter seasons with conditions that promote less water stress to produce seeds that can move farther away from their natal location. Whereas a longer growing season, which is more prone to water‐limitation on alvar habitats (increased DD5) leads to shorter dispersal distances with seeds remaining in their natal locations. However, in prairie populations where soils are deeper and there is more access to water, the growing season may extend as degree days increase. Here, *G. triflorum* has more time for active growth (Kulbaba et al., 2023) and can send its seeds farther. Recent evidence examining the phenological response of *G. triflorum* reflects these trends, where traits associated with initial establishment are under strong genetic control, but later life‐history stages are under stronger environmental control (Kulbaba et al., 2023).

The colonization history of isolated alvar habitats suggests that the strength and direction of selection for dispersal traits for grassland species, such as *G. triflorum*, isolated in alvar habitats could have changed over time (Catling & Brownell, 1995; Hamilton & Eckert, 2007). Previous research suggests that alvar habitats were likely colonized by grassland species during the warming Hypsithermal period ~5000 YBP as grasslands expanded (Hamilton & Eckert, 2007). However, following a cooling period, subsequent range contractions likely isolated a number of grassland species, including *G. triflorum*, on alvar habitats (Catling & Brownell, 1995; Hamilton & Eckert, 2007). While traits associated with long‐distance dispersal may have been selected for during initial colonization of alvar habitats, following isolation selection may act against traits associated with dispersal, as our results suggest. Furthermore, ecological specialization and local adaptation across alvar habitats may limit further gene flow, strengthening selection against dispersal (Van Den Elzen et al., 2016; Yoko et al., 2020). In order to determine the potential strength of selection in these two different habitats, reciprocal transplants would be required to tease apart potential confounding effect of having different soil conditions between these two habitats, as well as the respective role of the environmental variation from genetic differences, which reflect both heritable genetic differences and maternal effects, both of which have been shown to alter dispersal traits (Donohue, 1998; Galloway, 2005; Jacobs & Lesmeister, 2012; Yoko et al., 2020). While we have captured a snapshot of the relationship between environment and dispersal trait variation, we have observed that much of the variation can be explained by the maternal line, as well as variation caused by the environment at each site (large proportion of variation explained by the random effects). Temporal monitoring of trait variation within reciprocal transplant experiments will aid in understanding the role that plasticity and maternal effects play in maintaining variation in dispersal traits in response to seasonal environmental change.

There are substantial eco‐evolutionary consequences for differences observed in contemporary dispersal traits, such as those observed in *G. triflorum*. When considering the ability for diaspores to move, terminal velocity is critical (Wilson, 2000). This composite trait takes into account mass, morphology, and physical structures, such as hairs, to create differences in the length of time a diaspore can stay aloft in a column of air (Greene & Johnson, 1990; Matlack, 1987; Platt & Weis, 1977; Sheldon & Burrows, 1973). Terminal velocity plays an important role in a species' response to habitat fragmentation (Schleicher et al., 2011), and indirectly influences gene flow and the maintenance of connectivity across populations. In addition, we found that climate and habitat type altered another important dispersal trait, achene mass. This trait plays a role in establishment once individuals have dispersed, as larger propagules tend to have a higher survival probability during establishment (Moles & Westoby, 2004; Skarpaas et al., 2011). We find that achene mass and terminal velocity exhibit similar trends, as achene mass increases with increasing growing degree days in prairie habitats, but only slightly decreases with increased growing degree days in alvar habitats. When combined with terminal velocity, our results indicate that as climates warm and the number of growing degree days increases, *G. triflorum* populations in prairie habitats have the potential to disperse farther and may have an increased establishment probability, while alvar populations exhibit decreased dispersal and establishment potential.

In the landscapes over which *G. triflorum* occurs (i.e., continuous prairies vs. disjunct alvars), the spatial scale of habitat availability is highly relevant to selection on dispersal ability. For alvar versus prairie habitats, the frequency and location of suitable habitats may impact dispersal trait variation. According to the theory of island biogeography (MacArthur & Wilson, 1967), populations in isolated habitats may select against dispersal, particularly where long‐distance dispersal leads to reduced fitness as organisms disperse beyond the range of suitable habitats (Reluga & Shaw, 2015; Schenk, 2013; Shaw et al., 2019). Disjunct alvar habitats resemble oceanic islands, existing as open, isolated limestone barrens situated within a matrix of boreal forest. Similar to gypsum outcrops described by Van Den Elzen et al. (2016) and in increasingly patchy landscapes in Spain as described by Riba et al. (2009), the low frequency of disjunct alvar habitats relative to more continuous prairies will lead to strong selection against dispersal within alvar populations, as propagules that disperse longer distances likely land in unsuitable habitat. While selection may have shifted to favor reduced dispersal for isolated alvar populations, the same may not be true of prairie populations. Relatively continuous prairie habitats increase the likelihood that dispersing propagules will land in suitable habitats, contributing to selection for increased dispersal (Travis & Dytham, 1999). However, previous and ongoing agricultural conversion may increase the scale of isolation for populations within a matrix of inhospitable environments (Lark et al., 2018; Wimberly et al., 2018; Wright & Wimberly, 2013). Thus, increasing fragmentation across prairie environments has the potential to shift the direction of selection for dispersal traits in these historically continuous habitats. Our study remains an observational, spatially implicit examination of how dispersal traits change in relation to the environment. We encourage future work to explore how these altered traits might influence spatial recruitment patterns in open habitat patches within the landscape (Platt & Weis, 1977).

Climatic variation and shifting habitat suitability have the potential to rapidly alter dispersal potential, impacting predictions for connectivity and species' range shifts in response to global change (Kubisch et al., 2013). Where growing seasons are predicted to change, region‐specific dispersal potential in *G. triflorum* may become further reinforced (McGinn & Shepherd, 2003; Pryor et al., 2013; Wuebbles & Hayhoe, 2004). This may impact the evolutionary potential of populations, particularly those isolated alvar populations at the periphery of a species' range where reduced connectivity influences the maintenance of genetic variation needed to adapt to change (Jump & Peñuelas, 2005). Grasslands tend to be dominated by plants that disperse by wind (Lorts et al., 2008; Packard & Mutel, 1997). As climates warm, wind speed and turbulence patterns are expected to increase in the prairie region (Kling & Ackerly, 2020), which could further enhance dispersal in these more open, continuous systems. While our study is observational in nature, we encourage future studies to control for the effects of climate on plants from isolated and continuous habitats in order to parse out how dispersal is (or is not) changing. Understanding a species' ability to sustain an evolutionary response to changing conditions is necessary for the conservation of plant populations, and requires an evaluation of the interaction between climate and landscape structure to establish predictions for species distributions and connectivity under global change.

## AUTHOR CONTRIBUTIONS

**Lauren L. Sullivan:** Conceptualization (equal); data curation (equal); formal analysis (lead); investigation (equal); methodology (lead); project administration (equal); resources (equal); supervision (equal); visualization (lead); writing – original draft (lead); writing – review and editing (equal). **Zoe M. Portlas:** Investigation (equal); writing – review and editing (equal). **Kelsey M. Jaeger:** Investigation (equal); writing – review and editing (equal). **Mercedes Hoffner:** Investigation (equal); writing – review and editing (equal). **Jill A. Hamilton:** Conceptualization (equal); data curation (equal); formal analysis (supporting); funding acquisition (equal); investigation (equal); methodology (lead); project administration (equal); resources (lead); supervision (equal); writing – review and editing (equal).

### OPEN RESEARCH BADGES

This article has earned Open Data and Open Materials badges. Data and materials are available at data and code are available at GitHub during the review process: https://github.com/LLSullivan/GeumDispersal. Upon acceptance data and code will also be permanently stored at Dryad. https://github.com/LLSullivan/GeumDispersal (see above for plan for long term data and code storage).

## Data Availability

Data and code for analyses and figure creation can be found at Dryad https://doi.org/10.5061/dryad.3bk3j9kt0.

## References

[ece311231-bib-0001] Anderson, R. H. , Fuhlendorf, S. D. , & Engle, D. M. (2006). Soil nitrogen availability in tallgrass prairie under the fire‐grazing interaction. Rangeland Ecology & Management, 59(6), 625–631. 10.2111/05-088R2.1

[ece311231-bib-0002] Arshad, W. , Sperber, K. , Steinbrecher, T. , Nichols, B. , Jansen, V. A. A. , Leubner‐Metzger, G. , & Mummenhoff, K. (2019). Dispersal biophysics and adaptive significance of dimorphic diaspores in the annual *Aethionema arabicum* (Brassicaceae). New Phytologist, 221(3), 1434–1446. 10.1111/nph.15490 30230555 PMC6492137

[ece311231-bib-0003] Barton, K. (2018). MuMIn: Multi‐Model Inference. R package version 1.40.4 . https://cran.r‐project.org/package=MuMIn

[ece311231-bib-0004] Bates, D. , Machler, M. , Bolker, B. , & Walker, S. (2015). Fitting linear mixed‐effects models using lme4. Journal of Statistical Software, 67(1), 1–48.

[ece311231-bib-0005] Beaubien, E. , & Hamann, A. (2011). Spring flowering response to climate change between 1936 and 2006 in Alberta, Canada. BioScience, 61(7), 514–524. 10.1525/bio.2011.61.7.6

[ece311231-bib-0006] Beckman, N. G. , Aslan, C. E. , Rogers, H. S. , Kogan, O. , Bronstein, J. L. , Bullock, J. M. , Hartig, F. , HilleRisLambers, J. , Zhou, Y. , Zurell, D. , Brodie, J. F. , Bruna, E. M. , Cantrell, R. S. , Decker, R. R. , Efiom, E. , Fricke, E. C. , Gurski, K. , Hastings, A. , Johnson, J. S. , … Zambrano, J. (2020). Advancing an interdisciplinary framework to study seed dispersal ecology. AoB Plants, 12(2), 1–18. 10.1093/AOBPLA/PLZ048 PMC7179845 32346468

[ece311231-bib-0007] Beckman, N. G. , & Sullivan, L. L. (2023). The causes and consequences of seed dispersal. Annual Review of Ecology, Evolution, and Systematics, 2023(54), 2023. 10.1146/annurev-ecolsys-102320

[ece311231-bib-0008] Belcher, J. W. , Keddy, P. A. , & Catling, P. M. (1992). Alvar vegetation in Canada: A multivariate description at two scales. Canadian Journal of Botany, 70(6), 1279–1291.

[ece311231-bib-0009] Bernard‐Verdier, M. , Navas, M. L. , Vellend, M. , Violle, C. , Fayolle, A. , & Garnier, E. (2012). Community assembly along a soil depth gradient: Contrasting patterns of plant trait convergence and divergence in a Mediterranean rangeland. Journal of Ecology, 100(6), 1422–1433. 10.1111/1365-2745.12003

[ece311231-bib-0010] Bjorkman, A. D. , Myers‐Smith, I. H. , Elmendorf, S. C. , Normand, S. , Rüger, N. , Beck, P. S. A. , Blach‐Overgaard, A. , Blok, D. , Cornelissen, J. H. C. , Forbes, B. C. , Georges, D. , Goetz, S. J. , Guay, K. C. , Henry, G. H. R. , HilleRisLambers, J. , Hollister, R. D. , Karger, D. N. , Kattge, J. , Manning, P. , … Weiher, E. (2018). Plant functional trait change across a warming tundra biome. Nature, 562(7725), 57–62. 10.1038/s41586-018-0563-7 30258229

[ece311231-bib-0011] Brehm, A. M. , Mortelliti, A. , Maynard, G. A. , & Zydlewski, J. (2019). Land use change and the ecological consequences of personality in small mammals. Ecology Letters, 22, 1387–1395. 10.1111/ele.13324 31207017

[ece311231-bib-0012] Brownell, V. R. , & Riley, J. L. (2000). The Alvars of Ontario: Significant alvar areas in the Ontario Great Lakes region . Federation of Ontario Naturalists.

[ece311231-bib-0013] Catling, P. M. , & Brownell, V. R. (1995). A review of the alvars of the Great Lakes region: Distribution, floristic composition, biogeography and protection. Canadian Field‐Naturalist, 109(2), 143–171.

[ece311231-bib-0014] Cheptou, P.‐O. , Carrue, O. , Rouifed, S. , & Cantarel, A. (2008). Rapid evolution of seed dispersal in an urban environment in the weed Crepis sancta. Proceedings of the National Academy of Sciences of the United States of America, 105(10), 3796–3799. 10.1073/pnas.0708446105 18316722 PMC2268839

[ece311231-bib-0015] Corlett, R. T. , & Westcott, D. A. (2013). Will plant movements keep up with climate change? Trends in Ecology & Evolution, 28(8), 482–488. 10.1016/j.tree.2013.04.003 23721732

[ece311231-bib-0016] Damschen, E. I. , Baker, D. V. , Bohrer, G. , Nathan, R. , Orrock, J. L. , Turner, J. R. , Brudvig, L. A. , Haddad, N. M. , Levey, D. J. , & Tewksbury, J. J. (2014). How fragmentation and corridors affect wind dynamics and seed dispersal in open habitats. Proceedings of the National Academy of Science, 111(9), 3484–3489. 10.1073/pnas.1308968111 PMC3948294 24567398

[ece311231-bib-0017] Davis, M. B. , & Shaw, R. G. (2001). Range shifts and adaptive responses to quaternary climate change. Science, 292(5517), 673–679. 10.1126/science.292.5517.673 11326089

[ece311231-bib-0018] Delattre, T. , Baguette, M. , Burel, F. , Stevens, V. M. , Quénol, H. , & Vernon, P. (2013). Interactive effects of landscape and weather on dispersal. Oikos, 122(11), 1576–1585. 10.1111/j.1600-0706.2013.00123.x

[ece311231-bib-0019] Dickson, T. L. , Mittelbach, G. G. , Reynolds, H. L. , & Gross, K. L. (2014). Height and clonality traits determine plant community responses to fertilization. Ecology, 95(9), 2443–2452. 10.1890/13-1875.1

[ece311231-bib-0020] Donohue, K. (1998). Maternal determinants of seed dispersal in Cakile edentula: Fruit, plant, and site traits. Ecology, 79(8), 2771–2788. 10.2307/176516

[ece311231-bib-0021] Edelaar, P. , & Bolnick, D. I. (2012). Non‐random gene flow: An underappreciated force in evolution and ecology. Trends in Ecology & Evolution, 27(12), 659–665. 10.1016/j.tree.2012.07.009 22884295

[ece311231-bib-0022] Fahrig, L. (2017). Ecological responses to habitat fragmentation per se. Annual Review of Ecology, Evolution, and Systematics, 48(1), 1–23. 10.1146/annurev-ecolsys-110316-022612

[ece311231-bib-0023] Gajewski, W. (1958). Evolution in the genus Geum. Evolution, 13(3), 378–388. 10.2307/2406114

[ece311231-bib-0024] Galic, N. , Sullivan, L. L. , Grimm, V. , & Forbes, V. E. (2018). When things don't add up: Quantifying impacts of multiple stressors from individual metabolism to ecosystem processing. Ecology Letters, 21(4), 568–577. 10.1111/ele.12923 29460496

[ece311231-bib-0025] Galloway, L. F. (2005). Maternal effects provide phenotypic adaptation to local environmental conditions. The New Phytologist, 166(1), 93–99. 10.1111/j.1469-8137.2004.01314.x 15760354

[ece311231-bib-0026] Greene, D. F. , & Johnson, E. A. (1989). A model of wind dispersal of winged or plumed seeds. Ecology, 70(2), 339–347.

[ece311231-bib-0027] Greene, D. F. , & Johnson, E. A. (1990). The aerodynamics of plumed seeds. Functional Ecology, 4(1), 117–125.

[ece311231-bib-0028] Hamilton, J. A. , & Eckert, C. G. (2007). Population genetic consequences of geographic disjunction: A prairie plant isolated on Great Lakes alvars. Molecular Ecology, 16(8), 1649–1660. 10.1111/j.1365-294X.2007.03241.x 17402980

[ece311231-bib-0029] Hamilton, W. D. , & May, R. M. (1977). Dispersal in stable habitats. Nature, 269, 578–581.

[ece311231-bib-0030] Hargreaves, A. L. , Bailey, S. F. , & Laird, R. A. (2015). Fitness declines towards range limits and local adaptation to climate affect dispersal evolution during climate‐induced range shifts. Journal of Evolutionary Biology, 28(8), 1489–1501. 10.1111/jeb.12669 26079367

[ece311231-bib-0031] Harikrishna, B. , Dasgog, G. , & Patil, P. (2005). Effect of soil depths, N‐doses and its split applications on maize: I. Plant height, LAI and dry matter yield at different growth stages. Karnataka Journal of Agricultural Sciences, 18(2), 364–369. https://www.researchgate.net/publication/259492791

[ece311231-bib-0032] Harpole, W. S. , Ngai, J. T. , Cleland, E. E. , Seabloom, E. W. , Borer, E. T. , Bracken, M. E. S. , Elser, J. J. , Gruner, D. S. , Hillebrand, H. , Shurin, J. B. , & Smith, J. E. (2011). Nutrient co‐limitation of primary producer communities. Ecology Letters, 14(9), 852–862. 10.1111/j.1461-0248.2011.01651.x 21749598

[ece311231-bib-0033] Hernandez, J. O. , Naeem, M. , & Zaman, W. (2023). How does changing environment influence plant seed movements as populations of dispersal vectors decline? In Plants (Vol. 12, Issue 7). MDPI. 10.3390/plants12071462 PMC10097094 37050088

[ece311231-bib-0034] Higgins, S. I. , Flores, O. , & Schurr, F. M. (2008). Costs of persistence and the spread of competing seeders and sprouters. Journal of Ecology, 96, 679–686. 10.1111/j.1365-2745.2008.0

[ece311231-bib-0035] Jacobs, B. S. , & Lesmeister, S. A. (2012). Maternal environmental effects on fitness, fruit morphology and ballistic seed dispersal distance in an annual forb. Functional Ecology, 26(3), 588–597. 10.1111/j.1365-2435.2012.01964.x

[ece311231-bib-0036] Jump, A. S. , & Peñuelas, J. (2005). Running to stand still: Adaptation and the response of plants to rapid climate change. Ecology Letters, 8(9), 1010–1020. 10.1111/j.1461-0248.2005.00796.x 34517682

[ece311231-bib-0037] Katul, G. G. , Porporato, A. , Nathan, R. , Siqueira, M. , Soons, M. B. , Poggi, D. , Horn, H. S. , & Levin, S. A. (2005). Mechanistic analytical models for long‐distance seed dispersal by wind. The American Naturalist, 166(3), 368–381. 10.1086/432589 16224691

[ece311231-bib-0038] Kling, M. M. , & Ackerly, D. D. (2020). Global wind patterns and the vulnerability of wind‐dispersed species to climate change. Nature Climate Change, 10(9), 868–875. 10.1038/s41558-020-0848-3

[ece311231-bib-0039] Kokko, H. , & López‐Sepulcre, A. (2006). From individual dispersal to species ranges: Perspectives for a changing world. Science, 313(5788), 789–791. 10.1126/science.1128566 16902127

[ece311231-bib-0040] Kubisch, A. , Degen, T. , Hovestadt, T. , & Poethke, H. J. (2013). Predicting range shifts under global change: The balance between local adaptation and dispersal. Ecography, 36(8), 873–882. 10.1111/j.1600-0587.2012.00062.x

[ece311231-bib-0041] Kulbaba, M. W. , Yoko, Z. , & Hamilton, J. A. (2023). Chasing the fitness optimum: Temporal variation in the genetic and environmental expression of life‐history traits for a perennial plant. Annals of Botany, 132, 1–14. 37493041 10.1093/aob/mcad100 PMC10902883

[ece311231-bib-0042] Kuparinen, A. , Katul, G. , Nathan, R. , & Schurr, F. M. (2009). Increases in air temperature can promote wind‐driven dispersal and spread of plants. Proceedings of the Royal Society B: Biological Sciences, 276(1670), 3081–3087. 10.1098/rspb.2009.0693 PMC2817131 19515658

[ece311231-bib-0043] Kuznetsova, A. , Bruun Brockhoff, P. , & Haubo Bojesen Christensen, R. (2014). *lmerTest: Tests for random and fixed effects for linear mixed effect models* (*lmer objects of lme4 package*). http://cran.r‐project.org/package=lmerTest

[ece311231-bib-0044] Lark, T. J. , Larson, B. , Schelly, I. , Batish, S. , & Gibbs, H. K. (2018). Accelerated conversion of native prairie to cropland in Minnesota. Environmental Conservation, 1–8, 155–162. 10.1017/s0376892918000437

[ece311231-bib-0045] Lenser, T. , Graeber, K. , Cevik, Ö. S. , Adigüzel, N. , Dönmez, A. A. , Grosche, C. , Kettermann, M. , Quellhorst, S. M. , Mérai, Z. , Mohammadin, S. , Nguyen, T. P. , Rümpler, F. , Schulze, C. , Sperber, K. , Steinbrecher, T. , Wiegand, N. , Strnad, M. , Scheid, O. M. , Rensing, S. A. , … Leubner‐Metzger, G. (2016). Developmental control and plasticity of fruit and seed dimorphism in *Aethionema arabicum* . Plant Physiology, 172(3), 1691–1707. 10.1104/pp.16.00838 27702842 PMC5100781

[ece311231-bib-0046] Lentink, D. , Dickson, W. B. , van Leeuwen, J. L. , & Dickinson, M. H. (2009). Leading‐edge vortices elevate lift of autorotating plant seeds. Science (New York, N.Y.), 324(5933), 1438–1440. 10.1126/science.1174196 19520959

[ece311231-bib-0047] Lorts, C. M. , Briggeman, T. , & Sang, T. (2008). Evolution of fruit types and seed dispersal: A phylogenetic and ecological snapshot. Journal of Systematics and Evolution, 46(3), 396–404. 10.3724/SP.J.1002.2008.08039

[ece311231-bib-0048] MacArthur, R. H. , & Wilson, E. O. (1967). The theory of Island biogeography. Princeton University Press.

[ece311231-bib-0049] Matlack, G. R. (1987). Diaspore size, shape, and fall behavior in wind‐dispersed plant species. American Journal of Botany, 74(8), 1150–1160. https://about.jstor.org/terms

[ece311231-bib-0050] McGinn, S. M. , & Shepherd, A. (2003). Impact of climate change scenarios on the agroclimate of the Canadian prairies. Canadian Journal of Soil Science, 83(5), 623–630. 10.4141/s02-004

[ece311231-bib-0051] Moles, A. T. , & Westoby, M. (2004). Seedling survival and seed size: A synthesis of the literature. Journal of Ecology, 92(3), 372–383. 10.1111/j.0022-0477.2004.00884.x

[ece311231-bib-0052] Van Den Elzen, C. L. , LaRue, E. A. , & Emery, N. C. (2016). Oh, the places you'll go! Understanding the evolutionary interplay between dispersal and habitat adaptation as a driver of plant distributions. American Journal of Botany, 103(12), 2015–2018. 10.3732/ajb.1600312 27965241

[ece311231-bib-0053] Nelson, P. W. (1987). The Terrestrial Natural Communities of Missouri . Missouri Department of Conservation.

[ece311231-bib-0054] Packard, S. , & Mutel, C. F. (1997). The tallgrass restoration handbook: For prairies, savannas, and woodlands. Island Press.

[ece311231-bib-0055] Parmesan, C. , & Yohe, G. (2003). A globally coherent fingerprint of climate change impacts across natural systems. Nature, 421(6918), 37–42. 10.1038/nature01286 12511946

[ece311231-bib-0056] Platt, W. J. , & Weis, I. M. (1977). Resource partitioning and competition within a Guild of Fugitive Prairie Plants. American Naturalist, 111(979), 479–513. https://about.jstor.org/terms

[ece311231-bib-0057] Pryor, S. C. , Barthelmie, R. J. , & Schoof, J. T. (2013). High‐resolution projections of climate‐related risks for the Midwestern USA. Climate Research, 56(1), 61–79. 10.3354/cr01143

[ece311231-bib-0058] R Core Team . (2022). R: A Language and Environment for Statistical Computing .

[ece311231-bib-0059] Reluga, T. C. , & Shaw, A. K. (2015). Resource distribution drives the adoption of migratory, partially migratory, or residential strategies. Theoretical Ecology, 8(4), 437–447. 10.1007/s12080-015-0263-y

[ece311231-bib-0060] Renton, M. , Childs, S. , Standish, R. , & Shackelford, N. (2013). Plant migration and persistence under climate change in fragmented landscapes: Does it depend on the key point of vulnerability within the lifecycle? Ecological Modelling, 249, 50–58. 10.1016/j.ecolmodel.2012.07.005

[ece311231-bib-0061] Renton, M. , Shackelford, N. , & Standish, R. J. (2012). Habitat restoration will help some functional plant types persist under climate change in fragmented landscapes. Global Change Biology, 18(6), 2057–2070. 10.1111/j.1365-2486.2012.02677.x

[ece311231-bib-0062] Reschke, C. , Reid, R. , Jones, J. , Feeny, T. , & Potter, H. (1999). Conserving Great Lakes Alvars*. Final technical report of the international alvar conservation initiative* .

[ece311231-bib-0063] Riba, M. , Mayol, M. , Giles, B. E. , Ronce, O. , Imbert, E. , Van Der Velde, M. , Chauvet, S. , Ericson, L. , Bijlsma, R. , Vosman, B. , Smulders, M. J. M. , & Olivieri, I. (2009). Darwin's wind hypothesis: Does it work for plant dispersal in fragmented habitats? The New Phytologist, 183(3), 667–677. 19659587 10.1111/j.1469-8137.2009.02948.x

[ece311231-bib-0064] Rohrer, J. R. (1993). Geum. In Flora of North America. Editorial Committee (Ed.), Flora of North America North of Mexico. 20+ vols (Vol. 9, pp. 1–10). Oxford University Press.

[ece311231-bib-0065] Samson, F. , & Knopf, F. (1994). Prairie conservation in North America. BioScience, 44(6), 418–421.

[ece311231-bib-0066] Schaefer, C. A. , & Larson, D. W. (1997). Vegetation, environmental characteristics and ideas on the maintenance of alvars on the Bruce peninsula, Canada. Journal of Vegetation Science, 8(6), 797–810. 10.2307/3237024

[ece311231-bib-0067] Schenk, J. J. (2013). Evolution of limited seed dispersal ability on gypsum islands. American Journal of Botany, 100(9), 1811–1822. 10.3732/ajb.1300075 23997206

[ece311231-bib-0068] Schleicher, A. , Biedermann, R. , & Kleyer, M. (2011). Dispersal traits determine plant response to habitat connectivity in an urban landscape. Landscape Ecology, 26, 529–540. 10.1007/s10980-011-9579-1

[ece311231-bib-0069] Schneider, C. A. , Rasband, W. S. , & Eliceiri, K. W. (2012). NIH image to ImageJ: 25 years of image analysis. Nature Methods, 9(7), 671–675. 22930834 10.1038/nmeth.2089 PMC5554542

[ece311231-bib-0070] Seale, M. , & Nakayama, N. (2020). From passive to informed: Mechanical mechanisms of seed dispersal. New Phytologist, 225(2), 653–658. 10.1111/nph.16110 31403702

[ece311231-bib-0071] Sexton, J. P. , McIntyre, P. J. , Angert, A. L. , & Rice, K. J. (2009). Evolution and ecology of species range limits. Annual Review of Ecology, Evolution, and Systematics, 40(1), 415–436. 10.1146/annurev.ecolsys.110308.120317

[ece311231-bib-0072] Shaw, A. K. , D'Aloia, C. C. , & Buston, P. M. (2019). The evolution of marine larval dispersal kernels in spatially structured habitats: Analytical models, individual‐based simulations, and comparisons with empirical estimates. The American Naturalist, 3, 424–435. 10.1086/701667 30794444

[ece311231-bib-0073] Sheldon, J. C. , & Burrows, F. M. (1973). The dispersal effectiveness of the achene‐pappus units of selected Compositae in steady winds with convection. New Phytologist, 72(3), 665–675. 10.1111/j.1469-8137.1973.tb04415.x

[ece311231-bib-0074] Skarpaas, O. , SIlverman, E. J. , Jongejans, E. , & Shea, K. (2011). Are the best dispersers the best colonizers? Seed mass, dispersal and establishment in Carduus thistles. Evolutionary Ecology, 25, 155–169. 10.1007/s10682-010-9391-4

[ece311231-bib-0075] Soons, M. B. , Heil, G. W. , Nathan, R. , & Katul, G. G. (2004). Determinants of long‐distance seed dispersal by wind in grasslands. Ecology, 85(11), 3056–3068. 10.1890/03-0522

[ece311231-bib-0076] Sullivan, L. L. , Clark, A. T. , Tilman, D. , & Shaw, A. K. (2018). Mechanistically derived dispersal kernels explain species‐level patterns of recruitment and succession. Ecology, 99(11), 2415–2420. 10.1002/ecy.2498 30368793

[ece311231-bib-0077] Sullivan, L. L. , Michalska‐Smith, M. J. , Sperry, K. P. , Moeller, D. A. , & Shaw, A. K. (2021). Consequences of ignoring dispersal variation in network models for landscape connectivity. Conservation Biology, 35(3), 944–954. 32975336 10.1111/cobi.13640

[ece311231-bib-0078] Tardella, F. M. , Bricca, A. , Piermarteri, K. , Postiglione, N. , & Catorci, A. (2017). Context‐dependent variation of SLA and plant height of a dominant, invasive tall grass (*Brachypodium genuense*) in sub‐Mediterranean grasslands. Flora, 229, 116–123. 10.1016/j.flora.2017.02.022

[ece311231-bib-0079] Teller, B. J. , Campbell, C. , & Shea, K. (2014). Dispersal under duress: Can stress enhance the performance of a passively dispersed species? Ecology, 95(10), 2694–2698. 10.1890/14-0474.1

[ece311231-bib-0080] Thibault, E. , Soolanayakanahally, R. , & Keller, S. R. (2020). Latitudinal clines in bud flush phenology reflect genetic variation in chilling requirements in balsam poplar, *Populus balsamifera* . American Journal of Botany, 107(11), 1597–1605. 10.1002/ajb2.1564 33225462

[ece311231-bib-0081] Thomson, F. J. , Moles, A. T. , Auld, T. D. , & Kingsford, R. T. (2011). Seed dispersal distance is more strongly correlated with plant height than with seed mass. Journal of Ecology, 99(6), 1299–1307. 10.1111/j.1365-2745.2011.01867.x

[ece311231-bib-0082] Tilman, D. (1994). Competition and biodiversity in spatially structured habitats. Ecology, 75(1), 2–16. 10.2307/1939377

[ece311231-bib-0083] Travis, J. M. J. , & Dytham, C. (1999). Habitat persistence, habitat availability and the evolution of dispersal. Proceedings of the Royal Society B: Biological Sciences, 266(January), 723–728. 10.1098/rspb.1999.0696

[ece311231-bib-0084] Varshney, K. , Chang, S. , & Wang, Z. J. (2012). The kinematics of falling maple seeds and the initial transition to a helical motion. Nonlinearity, 25(1), C1–C8. 10.1088/0951-7715/25/1/C1

[ece311231-bib-0085] Visioli, G. , Sanangelantoni, A. M. , Conti, F. D. , Bonati, B. , Gardi, C. , & Menta, C. (2019). Above and belowground biodiversity in adjacent and distinct serpentine soils. Applied Soil Ecology, 133, 98–103. 10.1016/j.apsoil.2018.09.013

[ece311231-bib-0086] Volk, K. , Braasch, J. , Ahlering, M. , & Hamilton, J. A. (2022). Environmental contributions to the evolution of trait differences in *Geum triflorum*: Implications for restoration. American Journal of Botany, 109(11), 1822–1837. 10.1002/ajb2.16061 36151780

[ece311231-bib-0087] Wang, T. , Hamann, A. , Spittlehouse, D. , & Carroll, C. (2016). Locally downscaled and spatially customizable climate data for historical and future periods for North America. PLoS One, 11(6), 1–17. 10.1371/journal.pone.0156720 PMC4898765 27275583

[ece311231-bib-0088] Whittet, R. , Cavers, S. , Cottrell, J. , Rosique‐Esplugas, C. , & Ennos, R. (2017). Substantial variation in the timing of pollen production reduces reproductive synchrony between distant populations of *Pinus sylvestris* L. in Scotland. Ecology and Evolution, 7(15), 5754–5765. 10.1002/ece3.3154 28894569 PMC5586338

[ece311231-bib-0089] Wilson, J. D. (2000). Trajectory models for heavy particles in atmospheric turbulence: Comparison with observations. Journal of Applied Meteorology, 39(11), 1894–1912.

[ece311231-bib-0090] Wimberly, M. C. , Narem, D. M. , Bauman, P. J. , Carlson, B. T. , & Ahlering, M. A. (2018). Grassland connectivity in fragmented agricultural landscapes of the north‐central United States. Biological Conservation, 217, 121–130. 10.1016/j.biocon.2017.10.031

[ece311231-bib-0091] Wright, C. K. , & Wimberly, M. C. (2013). Recent land use change in the Western Corn Belt threatens grasslands and wetlands. Proceedings of the National Academy of Science, 110(10), 4134–4139. 10.1073/pnas.1215404110 PMC3593829 23431143

[ece311231-bib-0092] Wuebbles, D. J. , & Hayhoe, K. (2004). Climate change projections for the United States Midwest. Mitigation and Adaptation Strategies for Global Change, 9(4), 335–363. 10.1023/B:MITI.0000038843.73424.de

[ece311231-bib-0093] Yoko, Z. G. , Volk, K. L. , Dochtermann, N. A. , & Hamilton, J. A. (2020). The importance of quantitative trait differentiation in restoration: Landscape heterogeneity and functional traits inform seed transfer guidelines. AoB Plants, 12(2), 1–13. 10.1093/AOBPLA/PLAA009 PMC7112727 32257091

[ece311231-bib-0094] Zobel, M. , Moora, M. , & Herben, T. (2010). Clonal mobility and its implications for spatio‐temporal patterns of plant communities: What do we need to know next? Oikos, 119, 802–806. 10.1111/j.1600-0706.2010.18296.x

